# The measurement-based care to opioid treatment programs project (MBC2OTP): a study protocol using rapid assessment procedure informed clinical ethnography

**DOI:** 10.1186/s13722-022-00327-0

**Published:** 2022-08-19

**Authors:** Kelli Scott, John Guigayoma, Lawrence A. Palinkas, Francesca L. Beaudoin, Melissa A. Clark, Sara J. Becker

**Affiliations:** 1 Brown University School of Public Health, 121 South Main Street, Providence, RI 02903 USA; 2 Alpert Medical School of Brown University, 222 Richmond Street, Providence, RI 02903 USA; 3 University of Southern California, 669 West 34th Street, Los Angeles, CA 90089 USA

**Keywords:** Measurement-based care, Opioid use disorder, Opioid treatment programs, Mixed methods

## Abstract

**Background:**

Psychosocial interventions are needed to enhance patient engagement and retention in medication treatment within opioid treatment programs. Measurement-based care (MBC), an evidence-based intervention structure that involves ongoing monitoring of treatment progress over time to assess the need for treatment modifications, has been recommended as a flexible and low-cost intervention for opioid treatment program use. The MBC2OTP Project is a two-phase pilot hybrid type 1 effectiveness-implementation trial that has three specific aims: (1) to employ Rapid Assessment Procedure Informed Clinical Ethnography (RAPICE) to collect mixed methods data to inform MBC implementation; (2) to use RAPICE data to adapt an MBC protocol; and (3) to conduct a hybrid type 1 trial to evaluate MBC’s preliminary effectiveness and implementation potential in opioid treatment programs.

**Methods:**

This study will be conducted in two phases. Phase 1 will include RAPICE site visits, qualitative interviews (*N* = 32–48 total), and quantitative surveys (*N* = 64–80 total) with staff at eight programs to build community partnerships and evaluate contextual factors impacting MBC implementation. Mixed methods data will be analyzed using immersion/crystallization and thematic analysis to inform MBC adaptation and site selection. Four programs selected for Phase 2 will participate in MBC electronic medical record integration, training, and ongoing support. Chart reviews will be completed in the 6 months prior-to and following MBC integration (*N* = 160 charts, 80 pre and post) to evaluate effectiveness (patient opioid abstinence and treatment engagement) and implementation outcomes (counselor MBC exposure and fidelity).

**Discussion:**

This study is among the first to take forward recommendations to implement and evaluate MBC in opioid treatment programs. It will also employ an innovative RAPICE approach to enhance the quality and rigor of data collection and inform the development of an MBC protocol best matched to opioid treatment programs. Overall, this work seeks to enhance treatment provision and clinical outcomes for patients with opioid use disorder.

*Trial registration* This study will be registered with Clinicaltrials.gov within 21 days of first participant enrollment in Phase 2. Study Phase 1 (RAPICE) does not qualify as a clinical trial, therefore Phase 2 clinical trial registration has not yet been pursued because all elements of Phase 2 will be dependent on Phase 1 outcomes.

**Supplementary Information:**

The online version contains supplementary material available at 10.1186/s13722-022-00327-0.

Opioid overdoses are a public health emergency in the United States (US). In 2019, approximately 70% of prescription drug overdose deaths in the U.S. involved opioids [1]; preliminary data suggest that overdose rates have continued to rise during the COVID pandemic [2, 3]. To address this urgent public health issue, the gold standard, first-line evidence-based intervention is medication for opioid use disorder (MOUD), most commonly with methadone or buprenorphine [4–6]. MOUD is effective for reducing opioid use, improving treatment retention (when compared to placebo/no medication), and reducing the risk of overdose [4, 6]. However, MOUD is not sufficient for many patients, and treatment retention remains a challenge [7–10]. A meta-analysis of 18 randomized controlled trials revealed that only 51% to 63% of patients initiated on medication fully engage and remain in treatment for 3 to 6 months [7]. These retention numbers become as low as 43% across 6 to 12 months [7]. Such low retention rates increase the risk of lethal opioid overdose and heighten the need for evidence-based adjunctive psychosocial interventions [11].

Unfortunately, the uptake of adjunctive psychosocial interventions in opioid treatment programs (OTPs) that dispense methadone and buprenorphine has been extremely low [12, 13]. Counselors in OTPs have reported a myriad of challenges to implementing evidence-based manualized psychosocial interventions such as contingency management and cognitive behavioral therapy, including: limited time/resources to learn new models, lack of alignment with existing theoretical orientations, disagreements in the field regarding intensity and type of intervention, incompatibility with group therapy as a primary OTP treatment model, and beliefs that manualized interventions are too “rigid” or “inflexible” [14–17]. Thus, there is an unaddressed need for adjunctive psychosocial interventions that can be flexibly applied by OTP counselors in a group therapy format, delivered by counselors with an array of theoretical orientations, and scaled to accommodate the typical workflow in community-based OTPs.

Measurement-based care (MBC) is an evidence-based *intervention structure*, defined as a treatment element that guides selection of intervention content [18], that has the potential to meet the unique implementation needs of OTPs. MBC delivery with fidelity involves three key components: administration of a patient-reported outcome measure at the start of each session or appointment, counselor review of patient responses, and discussion of responses to identify focus areas and guide the remainder of the session [19]. MBC is intended to be employed routinely to enable monitoring of patient progress over time and assess the need for treatment modifications [20, 21]. This approach may be particularly well-suited for community-based OTPs for several key reasons. First, an MBC intervention structure can enhance treatment without requiring a counselor to change their theoretical orientation or typical approach to care, a common barrier in more complex interventions [18, 19]. Second, MBC has been employed effectively in group therapy, the primary counseling approach in many community OTPs, to monitor progress and guide treatment [19, 22, 23]. Third, in contrast to models such as contingency management that have demonstrated effectiveness in OTPs [24–26], MBC can be delivered with minimal costs because it does not require incentives. Fourth, MBC has been shown to enhance treatment engagement (of which treatment attendance is one component) in both mental health and substance use treatment through several hypothesized mechanisms, including enhancing the therapeutic alliance, improving client treatment involvement, and signaling the need for more accurate, client-centered changes to treatment [20, 21, 27–29]. Finally, MBC can be administered quickly, enhancing its feasibility for use in group counseling and in the context of the large patient census in many OTPs [30].

MBC has extensive evidence in the mental health literature as a structure for improving patient outcomes, especially for patients not progressing in treatment, based on data from correlational studies, randomized clinical trials, and cluster randomized trials [28, 31–33]. MBC has also demonstrated effectiveness in substance use treatment settings [34]. One 2012 study of patients seeking outpatient substance use treatment found that patients not making progress in treatment demonstrated reduced substance use and improved overall functioning when receiving adjunctive MBC [34]. A more recent 2019 implementation study [31] assessed the effects of MBC on treatment engagement among patients attending group substance use counseling at United States Department of Veteran’s Affairs clinics. Weekly MBC use was deemed feasible for implementation and provided valuable information to clinicians regarding risk for poor client treatment engagement [29]. Finally, addiction counselors have expressed openness to engaging in MBC within outpatient substance use treatment. In one qualitative study, outpatient counselors shared beliefs that MBC could enhance communication with their clients and expressed interest in measuring a variety of domains (e.g. coping skills, current substance use). However, counselors highlighted a need for MBC to be tailored to fit their setting, noting that MBC delivery would require high ease of use, personalized measurement for clients, and the ability to track change over time [35].

The growing evidentiary support for MBC has resulted in its recommendation as a best practice for substance use group counseling sessions in Veteran’s Administration clinics [22]. In this setting, patients complete a brief self-report measure, counselors review responses, and responses are used to guide group counseling content [22, 36, 37]. Use of MBC is also included in the evidence-based clinical practice guidelines for substance use disorder treatment (including opioid use disorder treatment) by the United States Department of Defense and Department of Veteran’s Affairs [38, 39]. Most recently, the Director of the National Institute on Drug Abuse and colleagues [40] recommended that MBC be applied within the OTP setting [36, 39–41], but to date no studies have evaluated the effectiveness or implementation of MBC in OTPs.

The Measurement-Based Care to Opioid Treatment Programs (MBC2OTP) Project addresses the need to advance the uptake of MBC in OTPs via three specific aims. Aim 1 is to inform MBC implementation via a novel mixed methods approach, Rapid Assessment Procedure Informed Clinical Ethnography (RAPICE) [42]. RAPICE embeds participant observation within front-line study team members and treatment providers engaged in rolling-out clinical procedures, combined with regular data review/analyses with an implementation science mixed methods expert consultant [42]. Aim 2 is to apply the RAPICE mixed-methods results to adapt an MBC assessment protocol for OTP use. Aim 3 is to simultaneously evaluate both effectiveness and implementation outcomes via a hybrid type 1 effectiveness-implementation trial. No a priori hypotheses were developed for Aims 1 and 2 given their exploratory nature and focus on development of an MBC protocol. Aim 3 will test the following hypotheses: (1)* Treatment engagement will increase across all sites following MBC electronic medical record integration*; (2) *Negative opioid urine screens will increase across all sites following MBC electronic medical record integration*; (3)* The extent to which counselors deliver MBC with fidelity will be positively associated with patient engagement in treatment sessions (both medication dosing and counseling appointments) and patient opioid abstinence across all sites.*

## Methods

### Study overview

The MBC2OTP Project has two phases. Phase 1 involves RAPICE methodology with eight OTPs, which combines rapid assessment procedures with clinical ethnography to facilitate partnership building and enhance the efficiency of data collection [42]. Phase 2 will consist of a pilot hybrid type 1 effectiveness-implementation trial in which the four OTPs selected during Phase 1 will engage in a collaborative design approach to integrate the refined MBC protocol into their workflow. This study is reported in line with SPIRIT guidelines (see SPIRIT Checklist in Additional file 1). Table 1 depicts a full study timeline.

**Table 1:** Phase 1 and 2 study timelines and associated activities

	Study period																			
	Year 1				Year 2				Year 3				Year 4				Year 5			
Phase 1/aims 1 and 2: RAPICE																				
Project launch	X	X	X																	
RAPICE stage 1: clinic site visits		X	X	X	X															
RAPICE stage 1: qualitative interviews			X	X	X															
RAPICE stage 1: quantitative surveys			X	X	X															
RAPICE stage 2: mixed methods data analysis					X	X	X													
RAPICE stage 3: MBC site selection							X	X												
RAPICE stage 3: MBC protocol adaptation								X	X											
Phase 2/aim 3: pilot MBC implementation																				
MBC EMR integration meetings												X	X							
MBC EMR integration site visits													X	X						
MBC workshop training and ongoing support															X	X				
MBC EMR patient outcome data extraction										X	X						X	X		
MBC EMR implementation outcome data extraction																	X	X		
MBC data analysis																		X	X	X

#### Phase 1: RAPICE

**Fig. 1:**
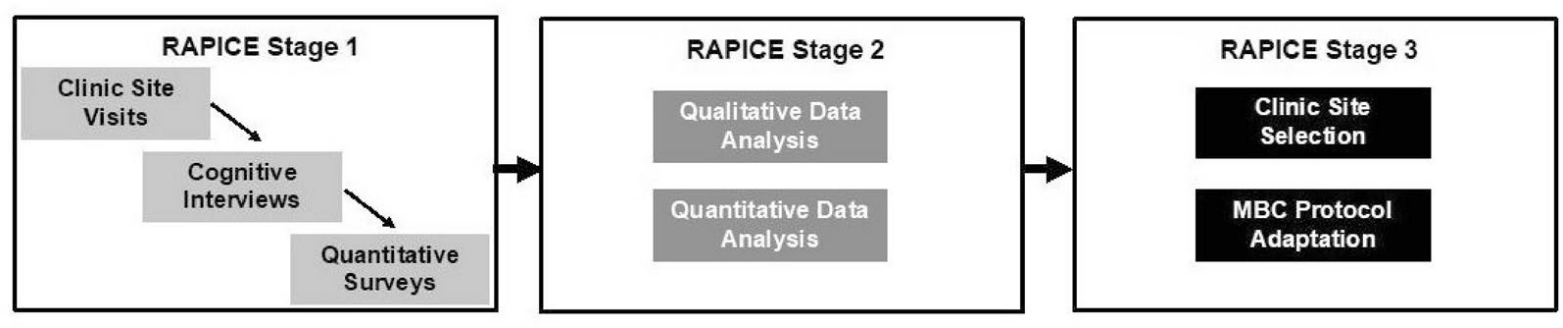
Phase 1 rapid assessment procedure informed clinical ethnography (RAPICE) stages and timeline

Research Phase 1 consists of three stages that take 3 months per OTP (see Fig. 1). OTPs will be recruited sequentially after a 3-month start-up phase: therefore, Phase 1 will take approximately 27 months. Stage 1 will involve site visits, informal and formal qualitative interviews, and quantitative surveys. Stage 2 will involve qualitative and quantitative data analysis by a multidisciplinary team. Stage 3 will involve using analyzed data to inform MBC assessment protocol adaptation. Completion of these stages will achieve several key objectives: development of community-engaged research partnerships with OTPs; evaluation of contextual factors impacting MBC implementation using mixed methods ethnography procedures; solicitation and utilization of counselor input to adapt an MBC assessment protocol for OTP treatment; and application of mixed methods data to inform site selection for MBC implementation [40].

##### Participant selection

Reflecting the multi-level nature of the study, participant selection will occur at three levels: OTP sites, OTP staff, and OTP patients.

OTP site selection: OTPs will be recruited from large multi-site organizations in New England. These organizations were selected because they: (1) have over 15 OTP locations serving New England, (2) dispense methadone and buprenorphine medications, and (3) provide a mix of group and/or individual counseling as an adjunct to pharmacotherapy.

Directors from large multi-site organizations have provided support for the participation of their OTPs and have provided contact information for a designated leader at each OTP. Researchers will contact each OTP’s designated leader to review an Organization Participation Agreement that details all components of study participation, including approval for the researchers to engage in site visits with each of the eight OTPs. The Organization Participation Agreement form is not intended to serve as a binding contract, but rather serves to document for organizational leaders what is being asked of their organization and what benefits their organizational staff will receive for participation.

OTP staff selection: Once a designated leader signs the Organizational Participation Agreement form, all other leaders, counselors, and staff (typically around 15 per OTP) at that location meeting eligibility criteria will be invited to participate in RAPICE activities. Up to two counselors will be recruited to serve as Participant Observers, six leaders and counselors will be recruited for RAPICE interviews, and up to 10 leaders, counselors, and staff will be recruited for RAPICE surveys per OTP. Contact information for all treatment counselors, leaders, and staff, including phone and email, will be provided by leaders at the participating OTPs. Study staff will then initiate contact to describe the study and obtain verbal informed consent. Counselors, leaders, and staff will be reassured that their decision whether or not to participate will not affect their employment in any way and that all feedback shared will remain confidential. In anticipation of OTP staff turnover, we anticipate the need to recruit additional staff (approximately 20% of total) over the course of the study to facilitate outcome data collection.

OTP leaders, counselors, and staff will need to be fluent in English to participate. Leaders must provide supervision to counselors delivering group counseling and have been employed at the OTP for at least 3 months. Leaders may include both administrative leaders (e.g. OTP Directors) or clinical leaders (e.g. clinical supervisors) and may provide either administrative supervision, clinical supervision, or both to counselors. Counselors must provide counseling in a group format and have been employed at the OTP for at least 3 months. Staff must have been employed at the organization for at least 3 months and work at the OTP in a non-counseling capacity (e.g., dosing nurses, security guards, front desk staff). Exclusion criteria are minimal to enhance generalizability and capture a range of stakeholder MBC preferences. Based on prior research conducted by our team within New England OTPs [14], we anticipate that participating leaders, counselors, and staff will be 83% Female, 83% White, 5% African American, 7% bi/multiracial, and 7% Hispanic/Latinx.

OTP patient selection: Each OTP will also be asked to refer one to two patients for qualitative interviews. Patients will be given consent-to-contact forms by OTP counselors or staff, which will grant permission for the research staff to contact them. Patients who complete the consent-to-contact form will be contacted via phone by research staff to complete the informed consent process. A waiver of documented consent has been obtained so that there is no written record linking patients to the study: patients will provide verbal informed consent to participate.

Patients must be fluent in English or Spanish, must have been newly inducted on medication for opioid use disorder within the past 6 months, and must be willing to complete a 30–60-min qualitative interview. Based on patient demographics from our team’s ongoing research in New England OTPs [43], we expect opioid treatment program patients to be 37% female, 88% White, 5% African American, 4% Asian, 2% bi/multiracial, and 8% Hispanic/Latinx.

##### RAPICE stage 1: clinic site visits and ethnographic observation

Researchers will visit each OTP site as participant observers to observe daily clinical procedures conducted by leaders, counselors, and staff; conduct informal conversations/interviews with OTP leaders, counselors, and staff, and attend full OTP team meetings. Participant observers are individuals who gather data by observing and engaging in the routine activities of a particular setting [42]. The goal of participant observation is to build rapport with the individuals in the community to be observed, as well as to facilitate comfort among those being observed so they can engage in typical activities without disruption [42]. Researchers can serve as participant observers, however, data quality is enhanced by also training treatment providers (i.e. the individuals actively providing care who have extensive knowledge of the setting) in ethnographic observation [42, 44]. As a result, the research team will also recruit OTP counselors to serve as participant observers for a 1-week period in order to enhance the quality of data collected regarding clinical procedures/workflows.

All participant observers will receive training in ethnographic methods by the study Principal Investigator (PI; KS) and the developer of the RAPICE approach (LP). Research team members and counselors will maintain written (daily procedures, staff meetings) memos and data logs regarding observations of daily procedures, staff meetings, and informal interviews to capture information gained from the site visits [42]. Information on these observations and interactions will be recorded through periodic jottings summarizing observations and more detailed field notes that will be updated each day. Field notes will also include impressions of events observed and exchanges with other counselors and staff, as well as preliminary interpretations of the significance of these events and exchanges. Each Participant Observer will then participate in a semi-structured debriefing interview with the mixed method consultant to clarify and expand upon information contained in jottings and field notes and provide a preliminary interpretation of their observations and interactions. Debriefs will be conducted using the Zoom conferencing platform, recorded, and transcribed for analysis. Participants will receive $75 for completion of the Participant Observer training, $20 for each submitted memo (up to $100), and $25 for completion of the debriefing interview.

##### RAPICE stage 1: qualitative interviews

Site visits will also include 30–60-min qualitative interviews with leaders, counselors, and patients to solicit feedback on the MBC protocol recommended by Marsden and colleagues (*N* = 4–6 per site; 32–48 total) [38]. OTP staff who do not provide counseling will not participate in this phase given the focus on obtaining feedback on MBC use in counseling. These interviews will be informed by best practice measure development methodology from the National Institutes of Health Patient Reported Outcomes Measurement Information System Initiative (PROMIS) [45]. The PROMIS Initiative recommends the development of assessment protocols through literature review, focus groups, and interviews [45]. Following PROMIS guidelines, qualitative interviews will be employed to evaluate leader, counselor, and patient preferences for adaptation of an MBC assessment protocol [40]. Participants will receive $50 for participation.

Participants will be presented with a list of MBC questions that map onto Marsden and colleagues’ recommendations (i.e. the DSM-5 opioid use disorder [OUD] criteria, see Table 2) [40]. Interviews will employ a respondent debriefing technique in which participants assess the perceived relevance and utility of each question. Participants will first complete each question as they would with a patient (for leaders and counselors) or counselor (for patients). The interviewer will then ask participants to consider how the information gathered from each question could inform their counseling approach/treatment plan. The debriefing technique will involve the use of scripted probes to inquire about the degree to which questions captured OTP assessment domains, clarity and meaning of questions, and appropriateness/fit of questions for use with OTP patients [46]. The interviewer will also ask the participant open-ended questions about preferences for including additional questions beyond the DSM-IV OUD criteria. Some example additional domains may include MOUD side effects, mental health symptoms, and treatment satisfaction. The interviewer will then ask each participant to consider the feasibility of the assessment protocol (i.e. the level of ease with which they could ask or complete MBC questions), how frequently MBC should be administered to establish an OTP-specific guideline, and preferred procedures for using MBC in group counseling sessions [40]. Finally, the interviews will include questions for leaders and counselors regarding treatment workflow and EMR documentation procedures. Qualitative interviews will be continued until saturation of themes is achieved related to MBC content, format, and administration considerations. Data collected in these qualitative interviews will inform questions asked in the subsequent quantitative surveys.

**Table 2:** Measurement-based care qualitative interview question table. Adapted from Marsden et al. [40]

Question	What does this mean to you?	Is this question clear? Relevant?	Would you recommend any wording changes? Recommend keeping or deleting?
1. Have you used opioids, sedatives, or cocaine in the past 3, 6, or 12 months?			
a. Yes			
b. No			
1a. If yes, how often have you used them?			
a. 5–6 times per week			
b. 3–4 times per week			
c. 2 times per week			
d. 1 time per week			
e. 1–3 times a month			
f. Less often			
1b. Did you inject any of these?			
a. Yes			
b. No			
2. Have you drank more than 4 (women) or 5 (men) standard drinks on a single occasion of 2 h or less in the past 3 months? A standard drink consists of…			
c. Yes			
d. No			
2a. If yes, how often did you drink this amount?			
a. 5–6 times per week			
b. 3–4 times per week			
c. 2 times per week			
d. 1 time per week			
e. 1–3 times a month			
f. Less often			
3. What typical dose of opioids do you take?			
4. Do you use any other opioids?			
a. Yes			
b. No			
What opioids?			
5. What is your motivation for seeking the effect of opioids?			
6. When do you typically experience symptoms of withdrawal?			
7. Where are you typically when you experience symptoms of withdrawal?			
8. Do you take any drugs to avoid or manage feelings of withdrawal?			
a. Yes			
b. No			
What drugs?			
9. Where do you typically obtain opioids?			
10. Do you use opioids with other people?			
a. Yes			
b. No			
What people?			
11. What thoughts and beliefs make you want to purchase opioids?			
12. Have you ever taken any actions to avoid using opioids?			
c. Yes			
d. No			
Were these actions successful?			
a. Yes			
b. No			
Why or why not?			
13. Have you ever experienced problems as a result of the time you have spent to get opioids?			
a. Yes			
b. No			
What kinds of problems?			
14. Have you ever had any negative experiences while using opioids or after?			
a. Yes			
b. No			
What were they?			
15. How strong is your typical urge to use opioids?			
a. 0—Not at all			
b. 1			
c. 2			
d. 3			
e. 4			
f. 5—Moderate			
g. 6			
h. 7			
i. 8			
j. 9			
k. 10—Extreme			
16. What types of situations or feelings cause you to want to use opioids?			
17. Have you ever been in any physically dangerous situations while using opioids (for example like driving or operating machinery)?			
a. Yes			
b. No			
What kinds of situations?			
18. Do you have any known physical health problems that are affected by your opioid use?			
a. Yes			
b. No			
If so, what are they? How does your opioid use make them worse?			
19. Has your opioid use ever impacted your personal roles at home, work, or school?			
a. Yes			
b. No			
If so, what impact has opioid use had on your roles?			
20. Has anyone close to you been affected by your opioid use?			
a. Yes			
b. No			
21 How often does your opioid use cause problems for you at home, work, or school, or with those close to you?			
a. 5–6 times per week			
b. 3–4 times per week			
c. 2 times per week			
d. 1 time per week			
e. 1–3 times a month			
f. Less often			
22. Have you reduced or given up any activities because of your opioid use?			
c. Yes			
d. No			
If so, what activities?			
22a. Do you think there’s any way to help you restart these activities?			
a. Yes			
b. No			

##### RAPICE stage 1: quantitative surveys

All eligible counselors, leaders, and staff (*N* = 8–10 per site; 64–80) across the eight OTPs will be emailed an invitation to complete a brief quantitative survey. We project 8–10 surveys will be returned per OTP given the typical OTP size of at least 15 full-time employees. Data will complement the Stage 1 qualitative interviews in line with a mixed methods structure of big qualitative and small quantitative data collection (QUAL + quan) for the function of convergence (i.e. to answer the same questions about factors impacting MBC implementation) [47].

Interested participants will review an electronic consent form via Qualtrics containing an overview of study procedures. Informed consent will be provided via an online Qualtrics form prior to starting the survey. Participants will receive $25 for survey completion. To reduce respondent burden, the entire survey will be under 50 items and should take only 10–15 min to complete. Measures will map onto the five dimensions of the Consolidated Framework for Implementation Research (CFIR), a comprehensive framework that considers multiple determinants of implementation [48]. The five key dimensions and specific validated measures used will be: Intervention Characteristics (e.g. relative advantage, adaptability, trialability)—Perceived Characteristics of Intervention Scale (20-items, alpha = 0.67–0.95) [49]; Outer Setting (e.g. external policies and incentives)—Texas Christian University Survey of Organizational Functioning (adapted 7-item version) [50]; Inner Setting (e.g., implementation climate)—Implementation Climate Scale (6-items; alpha = 0.89) [51]; Individual Characteristics (e.g., knowledge and beliefs about the intervention)—Monitoring and Feedback Attitudes Scale (14 items, alpha = 0.87) [52]; and Implementation Process (e.g., ideal process for implementing MBC)—Scale (5 items) to be developed based on additional information gained from responses to RAPICE interviews regarding typical OTP workflows and counselor preferences for MBC administration. We opted to develop the Implementation Process questions based on information from the qualitative interviews to ensure that process questions directly reflected OTP workflows (e.g. what job roles would be able to administer MBC, how frequently, and when during a typical treatment visit).

##### RAPICE stage 2: pragmatic mixed methods data analysis

A multidisciplinary team consisting of the investigators and a leadership representative from each of the community OTPs will review and analyze the site visit, qualitative interview, and quantitative survey data. The investigators selected a combination of an immersion/crystallization technique and thematic content analysis to facilitate rapid, less resource-intensive triangulation of the data. The immersion/crystallization technique involves reviewing and reflecting on site visit logs and informal interviews, preparing descriptive memos identifying topics and themes, and discussing commonalities in themes to form overall data impressions [42, 53].

Thematic content analysis will involve five steps consisting of two independent coders (a PhD-level psychologist and trained research assistant): (1) independent review of the qualitative interview transcripts to identify possible themes/codes; (2) integration of identified codes into a codebook of both a priori (due to structured interview nature of qualitative interviews) and emergent themes through consensus; (3) application of identified codebook to each of the 32–48 interview transcripts using NVivo 12 software [54]; (4) weekly evaluation of inter-rater reliability using a reflexive team analysis approach to identify all coding discrepancies and achieve 100% consensus through discussion [55, 56]; and (5) queries of the data to identify common a priori and emergent themes [42]. In the event that the two coders do not achieve consensus, a third coder (a PhD-level psychologist) will make final coding decisions. Thematic content analysis will identify emergent themes regarding both MBC preferences and implementation, including: counselor views on OTP progress assessment/MBC best practices, preferences regarding MBC content/need for additional questions, appropriateness and meaning of MBC questions, appropriateness/fit of questions for use in community OTPs, and preferred MBC administration frequency. To facilitate convergence of RAPICE qualitative data with quantitative surveys, the investigators will compute total and subscale scores for each of the quantitative surveys administered [47]. These scores will then be averaged to obtain average site CFIR domain scores.

##### RAPICE stage 3: MBC assessment protocol adaptation and site selection

After consensus has been achieved regarding core themes from RAPICE Stages 1 and 2, the MBC assessment protocol will be adapted. Measure adaptation will likely include modifications to the MBC content including addition of new questions, revision of question wording, and modification or addition of follow up probing questions. Researchers and community partners will also review mixed methods data to inform how frequently (i.e. weekly, biweekly) counselors will deliver MBC with patients, and how often implementation support (i.e., supervision) will be provided. This process will result in the establishment of an MBC assessment protocol that will delineate methods for its administration in group counseling sessions, documentation in the health record, and supervision procedures [31]. The adapted MBC assessment protocol will be used for implementation of the pilot trial.

The final step of the RAPICE methodology will be the selection of OTPs for the Phase 2 pilot MBC trial [57]. Researchers and community partners will review the RAPICE qualitative data along with average site scores on the quantitative surveys. Researchers will then select the four OTPs with the highest implementation potential for Phase 2 participation (i.e. highest ratings of attitudes about MBC, readiness to change, organizational climate, and workflow facilitative of MBC integration) [57].

#### Phase 2: pilot MBC hybrid effectiveness implementation trial

The objective of Phase 2 is to evaluate the effectiveness and implementation potential of MBC via a pilot hybrid type 1 MBC effectiveness-implementation trial with four OTPs. This phase will last approximately 33 months. Although MBC has extensive evidence in mental health and some substance use settings, evidence is limited in OTPs. Thus, consistent with best practices in a Type I hybrid effectiveness-implementation trials [58], this pilot study will prioritize data on effectiveness while simultaneously gathering implementation data.

##### Participant selection

Leaders and counselors will be actively recruited, and patient records will be extracted from the electronic medical record. The Phase 1 eligibility criteria will be used to determine eligibility of counselors and leaders (see Phase 1. Participants). Patient records will be included in data extractions if the patients were receiving methadone or buprenorphine along with group counseling; and were inducted on medication for opioid use disorder within 6 months of MBC integration. We anticipate that the demographics of the Phase 2 participants will be similar to those in Phase 1.

##### MBC delivery

**Fig. 2:**
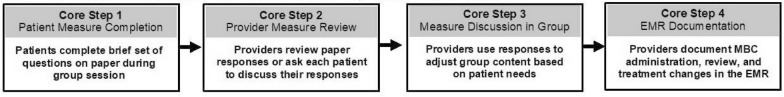
Measurement-based care core delivery steps for group counseling

The MBC protocol will be adapted based on RAPICE results. Anticipated MBC core delivery steps are presented in Fig. 2. The process, which aligns with MBC as delivered in group substance use treatment in the Veteran’s Administration [22, 36–38], will begin with the counselor administering MBC questions at the start of each group session via paper/pencil response forms. The counselor will then review each patient’s responses confidentially and use the responses to guide the agenda for the group session. For example, counselors may use responses to prompt group discussion about symptoms present for multiple group members, shift the session focus to target patient needs, discuss specific responses with individual group members during or following the group session, or use MBC responses to flag patients for follow up individual counseling or case management (i.e. treatment plan changes). Counselors may also use information regarding trends in group members’ MBC responses over time to highlight areas of improvement during the group session and plan group content for future sessions (e.g. focusing on specific skills or topics). Following each group session, counselors will document MBC delivery and responses in the health record to enable monitoring of change over time, in accordance with the MBC integration plan described below.

##### MBC integration

Informed by existing OTP workflow and health record documentation data gathered via RAPICE, researchers will meet with relevant leaders and information technology specialists at each of the OTPs to integrate the adapted MBC protocol into the workflow. Similar to Phase 1, each OTP will sign an Organization Participation Agreement outlining Phase 2 activities that will include an agreement to make electronic medical record changes to integrate MBC and extract de-identified data. At the beginning of the integration process, researchers will complete a second round of clinic site visits focused on integration of MBC into both group counseling and documentation procedures using collaborative research design principles [59–61]. The collaborative design approach will include meetings with leaders, counselors, and associated information technology specialists at each site to evaluate MBC documentation needs. OTPs provide a wide range of group counseling services covering numerous topics (e.g. recovery support, Cognitive Behavioral Therapy), and we anticipate that some patients may attend multiple groups each week. As a result, collaborative meetings will also focus on identifying which groups would be most appropriate for MBC integration. Informed consent will be obtained from all leaders and counselors participating in integration meetings. Up to 40 (approximately 8 to 10 per site) participants will be invited to contribute to meetings, each of which will be will be compensated $50.

The multi-site OTP organizations participating in this study use electronic medical record systems that can be modified by adding new templates, checklists, and/or dot phrases to facilitate documentation. Investigators will meet with appropriate health data specialists to determine an MBC documentation plan, which will be followed by changes to the electronic medical record interface based on feasibility and counselor needs. These electronic medical record changes may vary from a full, automated integration of MBC into provider documentation to the addition of simple dot phrases or note templates. Meetings with health data specialists will also involve development of methods for the OTPs to extract fully deidentified MBC outcome data from the electronic medical record [59, 61].

Once MBC has been integrated into the electronic medical record, OTP counselors will be invited to complete a 4-h MBC training workshop led by the investigative team. In addition, ongoing support (e.g. supervision) will be offered to counselors based on Phase 1 RAPICE feedback. Shortly after the workshop, counselors will begin using MBC and documenting all three steps (i.e., administration, review of responses, discussion with patients) in the electronic medical record.

##### Measures

All effectiveness and implementation outcome data collected in Phase 2 will be fully deidentified data extracted from the medical record. Counselor data will only be extracted for those who completed the MBC workshop, whereas patient data will only be extracted for those treated by counselors who completed the workshop. Patient treatment engagement data and opioid abstinence will be collected monthly across 6 months prior to and post MBC integration to evaluate preliminary effectiveness and implementation. Counselor MBC exposure and fidelity will be assessed post MBC integration only. Demographic data will also be extracted for all counselors and patients (e.g. age, biological sex, race, ethnicity). Finally, information about prior treatment at the OTP (i.e. patients who received care previously but came in again as new patients), patient MOUD type and dose, and patient DSM diagnoses (including other substance use or mental health disorders) will be extracted.

Patient treatment engagement: The primary MBC effectiveness outcome measure is patient treatment engagement, which we have broadly defined to encompass both completion of methadone/buprenorphine dosing visits and attendance at counseling/psychosocial intervention appointments. Approximately 20 charts per site (*N* = 80) will be randomly selected from new patients that entered treatment over the 6 months prior to MBC integration. An additional separate set of 20 new patient charts per site (*N* = 80) will be extracted to evaluate treatment engagement in the 6 months following integration. Charts will be reviewed to identify the proportion of treatment visits attended (i.e. MOUD dosing visits and individual/group counseling appointments) out of total visits scheduled for each patient, as well as the total number of weeks each patient was retained in treatment.

Patient opioid abstinence: The secondary MBC effectiveness outcome measure is patient opioid abstinence. The same charts extracted for treatment engagement will also be reviewed for opioid abstinence. Charts will be reviewed to identify the proportion of negative opioid urine screens for each patient monthly across 6 months prior to and post MBC integration. We anticipate that some patients in our chart sample will discontinue treatment (e.g. due to discharge or leaving the OTP) during the 6 months prior to and post MBC integration. For patients who discontinue treatment, opioid abstinence will be calculated as the proportion of negative urine screens for each month that they were present in the program. We will include an indicator in the data set to denote that urine drug screen data was unavailable for the months following patient treatment discontinuation.

Counselor MBC exposure: The primary MBC implementation outcome measure is monthly counselor MBC exposure. Counselor-level exposure refers to the number of group counseling sessions each month where MBC was administered divided by the total number of group sessions completed each month by OTP counselors (i.e. total opportunities to use MBC) [62]. The total number and dates of monthly completed group counseling sessions delivered by counselors who attended the MBC workshop. will be extracted from the EMR at all four sites. Documentation from each of these counseling sessions will be reviewed to determine whether or not MBC was recorded. Monthly MBC exposure values will be computed by dividing the total number of sessions with MBC documentation by the total number of sessions completed across each of the 6 months post MBC integration.

Counselor MBC fidelity: The secondary MBC implementation outcome measure is monthly counselor MBC fidelity. Counselor MBC fidelity refers to the degree to which counselors use MBC according to the established protocol (i.e. administering the questions, reviewing responses, discussing responses with patients in group counseling). Fidelity data will be computed as a categorical variable for completed group counseling sessions delivered by counselors who attended the MBC workshop across 6 months post integration. Counselors will receive a “1” if they documented MBC administration, a “2” if they documented administration and response review or administration and MBC discussion, and a “3” if they documented administration, response review, and MBC discussion with the patient. All attended counseling sessions without MBC documentation will receive a “0” [19].

##### MBC effectiveness and implementation outcome data analysis

Descriptive statistics will be explored (means, standard deviations, ranges) for all outcome variables to determine preliminary MBC effectiveness and implementation across all sites. Mean comparisons will test MBC effectiveness and implementation hypotheses. Specifically, *t*-tests will be conducted to compare average pre- and post-MBC treatment engagement proportions/number of weeks of MOUD and counseling attended and opioid abstinence proportions.

Additional exploratory analyses will employ Multi-Level Modeling to identify the impact of MBC fidelity on patient treatment engagement/abstinence as well as predictors of MBC exposure and fidelity at both the individual counselor and OTP level while accounting for data nested within OTPs. Predictors of patient treatment engagement/abstinence to be explored include biological sex, gender identity, age, race, ethnicity, prior MOUD receipt at the OTP, MOUD type and dose, and number of co-occurring disorders. Predictors of MBC exposure and fidelity to be explored at the counselor level include biological sex, gender identity, age, race, ethnicity, years employed at the OTP, and attitudes toward MBC. Predictors to be explored at the OTP level include organizational functioning and implementation climate (measured in Phase 1; see Phase 1 Quantitative Surveys). Missing data will be analyzed to evaluate percent missingness, and analyses will employ multiple imputation methods in the event of substantial missing data.

##### Sample size and power considerations

For Phase 2, previous studies of MBC have demonstrated small to large treatment effect sizes (Hedges’ *g* ranges from 0.10 to 0.53; Cohen’s *d* ranges from 0.18 to 0.50) [63]. A small to medium effect size in this study for either treatment engagement or opioid abstinence, paired with evidence of counselor acceptability and feasibility from the Phase 1 RAPICE, would indicate preliminary support for a larger trial of MBC in opioid treatment programs. The Phase 2 sample size of 160 (*N* = 80 new patient charts pre-MBC EMR integration and 80 charts post-EMR integration) is powered to detect a small to medium effect size of 0.39 using a basic pre- to post-test design. The detected MBC effect size in this study will be used to calculate post-hoc power analyses to determine the number of patients required for a fully-powered, two-group (MBC versus services as usual) effectiveness-implementation trial. For example, a sample size of 484 patients would be required to detect small effect sizes (*d* = 0.3), while a smaller sample of 176 patients would be required to detect medium effect sizes (*d* = 0.5). For large effect sizes, a sample of 70 patients would be required (*d* = 0.8).

#### Research ethics approval, data management, and dissemination

##### Ethics approval

This study is currently beginning Phase 1 RAPICE data collection. All RAPICE data collection procedures have received ethics approval from the Brown University Institutional Review Board. Any required protocol amendments throughout the study timeline will be reviewed and approved by the Institutional Review Board prior to implementation.

##### Data protections

The risk of breach of confidentiality is low considering all demographic information, qualitative data, and quantitative surveys collected from treatment counselors, leaders, staff, or patients will be stripped of any private health information and identified by numeric codes. Potential risk from breach of confidentiality will be minimized by strictly adhering to the guidelines for research outlined by the Brown University Institutional Review Board. This will include identifying participant research data by numeric identifier only and maintaining any records containing potentially identifying information separate from any research data. Physical research data will be kept in a locked file accessible only to research team and all electronic data will be password protected. Interview recordings will only be listened to by research team members and will be erased after being transcribed.

##### Data safety and monitoring

The project will be monitored by the study investigators on an ongoing basis to ensure the safety of study participants. Data collection in Phase 1 is brief in duration, harmless, and involves non-sensitive topics that pose no risks to subjects, as all data collection will be inquiring about treatment counselor, leader, staff, and patient perceptions of MBC. Data collection in Phase 2 is fully de-identified data extracted from the electronic medical record. In the unlikely scenario of an adverse event/serious adverse event observed by community partners or the study research assistant, the PI will be immediately notified. Adverse events/serious adverse events will be reported to the Brown University IRB in writing within 1 week. Serious adverse events will be reported to the National Institute on Drug Abuse Project Officer within 24 h. In the event of an adverse event, the investigators will integrate additional study safeguards recommendations from the Institutional Review Board. For any serious adverse events, data collection will be discontinued immediately upon notification.

##### Access to data

Data access will be limited to the study team during active data collection. Data will be available upon request from the study PI for both study phases at the conclusion of data collection. The full study protocol and statistical code will also be made available upon request from the study PI.

##### Data dissemination plan

Study activities in Phase 2 meet National Institutes of Health criteria for a clinical trial and therefore will follow a clear plan for dissemination of clinical trial information. The PI will register the clinical trial within 21 days of enrollment of the first participant in Study Phase 2. Clinical trial registration was not pursued during Phase 1 as all elements of Phase 2 (i.e. participating sites, MBC protocol) are dependent on the outcomes of RAPICE data collection. The investigators will also widely disseminate study findings via publication in peer-reviewed journals, presentation at international scientific conferences, and collaborative data meetings with participating OTPs.

## Discussion

This protocol paper describes the MBC2OTP Project, a two-phase pilot hybrid type 1 effectiveness-implementation study that aims to develop and implement an MBC protocol within OTPs providing medication for opioid use disorder to patients in New England. Recent increases in opioid overdose deaths, combined with an immense need to improve the reach and effectiveness of interventions for opioid use disorder, call for flexible evidence-based structures like MBC to enhance treatment [1, 7–10]. This study will employ RAPICE, a novel approach to building community partnerships and collecting mixed method data, to develop and test the effectiveness and implementation potential of an MBC protocol that is driven by the needs of OTP leaders, counselors, staff, and patients. This work aligns with prior literature highlighting the importance of designing MBC to fit the needs of addiction counselors and substance use treatment settings [35].

There are several key design considerations guiding this study that raise potential limitations as well as strengths. First, this study is a pilot trial and therefore has limited power to detect MBC’s effectiveness and implementation. It also relies on a pre- post- MBC integration outcome assessment design instead of a more traditional concurrent comparison of MBC versus treatment as usual. Finally, fidelity data for this study rely on provider self-report within the electronic medical record rather than objective fidelity monitoring. We opted to engage in a pilot trial rather than a full powered trial given the limited prior work evaluating MBC in community OTP settings. We aim to use RAPICE as a novel methodology to develop both the content of an MBC measure for OTP use as well as to inform the specific MBC protocol for this setting. We selected the pre- post- outcome assessment design and a simplified fidelity monitoring approach to enable data collection and pilot testing at a greater number of sites, thereby enhancing the generalizability of the MBC protocol and enabling more counselors to implement MBC. If promising, our future studies will aim to simultaneously assess MBC’s effectiveness and implementation in a fully powered hybrid type II effectiveness-implementation trial that includes more objective fidelity monitoring approaches (e.g. provider audio recordings, observation of MBC delivery).

Second, our study design focuses on the implementation of MBC within group counseling for new patients initiating treatment. While MBC has been traditionally studied in the context of individual counseling [21, 64], more recent work within the Department of Veteran’s Affairs has found that MBC can be feasibly and effectively delivered in the group context [29]. Group counseling was selected for this study as it is the primary treatment approach employed in OTPs given large patient numbers and a high demand for counseling services [65]. Newly inducted patients were selected as the study population for the preliminary MBC effectiveness outcome because newly inducted patients are most at risk for relapse [7–9].

Finally, the focus on high implementation potential sites was selected to enhance the potential for collection of high-quality MBC outcome data to evaluate MBC’s preliminary effectiveness in OTPs. A key goal of this study is to identify the acceptability and potential barriers to MBC implementation, even among these high potential OTPs. If data are promising, future work will focus on evaluating MBC implementation at low implementation potential sites to identify optimal implementation strategies for enhancing MBC scale up in that context. If significant MBC implementation challenges emerge, future work will instead focus on further development of an MBC protocol and implementation strategy to address these barriers. Overall, this study aims to engage in the essential foundational work needed to facilitate widespread scale-up of MBC to community OTPs, facilitate counselor monitoring of patient treatment progress, and potentially reduce the burden of disease associated with opioid use disorder. The MBC2OTP Project is among the first studies to take forward recommendations by Dr. Nora Volkow and colleagues to study MBC in OTPs [40], employ the cutting edge RAPICE methodology for collecting mixed methods data, and test the preliminary effectiveness and implementation of MBC in community OTP settings.

## Supplementary Information

**Additional file 1.** SPIRIT 2013 Checklist.

## Data Availability

Data collection for the study is just beginning and therefore data are available only to the research team at this time. Upon completion of data collection, all study data will be available on reasonable request from the corresponding author KS. The data will not be made publicly available due to them containing information that could compromise research participant consent.

## Abbreviations

MBC: Measurement based care
MOUD: Medication for opioid use disorder
OTP: Opioid treatment program
RAPICE: Rapid Assessment Procedure Informed Clinical Ethnography
PI: Principal investigator
